# Correlation of screen exposure to stress, learning, cognitive and language performance in children

**DOI:** 10.1007/s00787-024-02593-6

**Published:** 2024-10-23

**Authors:** Andrea Hahnefeld, Monika Fink, Saskia Le Beherec, Marie Anna Baur, Katharina Bernhardt, Volker Mall

**Affiliations:** 1 Chair of Social Pediatrics, TUM School of Medicine, Technical University of Munich, Munich, Germany; 2 Kbo Kinderzentrum, Heiglhofstrasse 65, 81377 Munich, Germany; 3 German Center for Child and Adolescent Health (DZKJ), Partner Site, Munich, Germany

**Keywords:** Screen exposure, Children, Stress, Cortisol, Learning, Language

## Abstract

The omnipresence of mobile screens and convenience to operate them has led to increased screen time for young children whereas the sequelae of prolonged exposure are not known yet. 70 refugee children (RG) and 111 children of a clinical comparison group (CG) from a help-seeking population (age: M = 5.10; SD = 1.11; range 3.00–6.97 years) were assessed concerning their amount of daily screen exposure time in relation to parental education and distress. Salivary cortisol was collected as a marker for biological stress and children were tested concerning learning performance, non-verbal IQ and vocabulary with the Kaufmann Assessment Battery for Children (KABC-II). Language skills were assessed in educator rating. The amount of children’s screen exposure was negatively related to parental education and positively to distress. In the CG, higher amounts of screen time were associated with elevated cortisol levels and lower learning scores. On both measures, the RG and CG only differed in the condition of screen time less than one hour/day, for higher amounts of screen time the CG approached the more problematic scores of the RG. Whereas in the whole sample the amount of screen time was negatively correlated to language performance, it was not correlated to non-verbal IQ-scores. As a higher amount of media exposure in our clinical comparison group is associated with elevated biological stress, decreased learning and lower language performance, it should be classified as a relevant environmental factor and regularly considered in clinical assessments of children and therapeutical interventions, especially in vulnerable subgroups. German clinical trials register, registration number: DRKS00025734, date: 07–23-2021.

## Introduction

Within the past few years, the omnipresence of mobile devices like smartphones and tablets has led to increased periods of screen exposure, which also affects children [1–3]. Further, the intuitively designed surfaces with touch-screens and voice-input make these devices easy to operate, lowering the starting age of unsupervised use [4, 5]. A recent survey shows that the average time of screen exposure in preschool children is more than 2 h per day [1]. This amount highly exceeds expert recommendations [6, 7] as the use of electronic media in early childhood reduces the quantity and quality of interaction between child and parent [8] and is reported to have diverse negative effects on language [9–18] and communication skills [19] as well as cognitive and executive functions [10, 18]. There are discrepant meta-analytic results reporting positive associations with educational media, respective no overall association between vocabulary assessment and media exposure in the home environment [20]. Positive effects are more pronounced in experimental studies, as specifically designed educational media have been shown to enhance word-learning in children [20]. Apart from special media developed for the purpose of word learning or language enhancement in a social-communicative context, screen time does not only reduce opportunities to learn the native language by interacting directly with other people, but also the time to have comforting interactions for the child with the primary caregivers during a period of high neuro-developmental plasticity. In addition, unsupervised screen access might expose the child to inadequate contents [1, 5], which are likely to elevate the overall stress level and interfere with the decline of stress hormones during daytime towards the evening [21]. This is in line with reports of sleep disturbances for children with high screen exposure times [22, 23]. Symptom load and sleeping behavior for young children are usually assessed in parent ratings. As these might be skewed by expectations and the parents’ own well-being and involvement [21], corresponding studies with associations of objectively assessed biological markers for children’s stress level are needed. Findings from large-scale studies suggest that, in general, elevated afternoon and evening levels of cortisol are associated with exposure to cumulative stress in early childhood [24–26].

Further, various literature from the recent years has demonstrated how different context factors play a crucial role not only for children’s circadian rhythm and development, but also for the families’ life and their media consumption habits [5, 18, 27]. As low socio-economic status, high maternal distress and low parental education do influence children’s health and development by themselves [28, 29] and are also associated with higher amounts of screen media exposure [4, 29, 30], corresponding factors might potentiate in populations at risk [15, 29]. In contrast, especially vulnerable groups, e.g. with developmentally delayed children, low socio-economic or migration background are harder to reach and less easy to involve in research [31] as many assessments on associations between screen use and child development have been conducted in samples with highly educated parents [10, 32].

The aim of this study is to analyze how the amount of screen exposure is related to the child’s stress level and learning, cognitive and language performance especially in populations of children with multiple risk factors like developmental delays, low parental education, multilingual contexts, migration and refugee background. We hypothesize higher salivary cortisol levels during the second half of the day as well as lower learning, cognitive and language performance in children with higher amounts of screen exposure.

## Methods

This study is part of the INterCuLtUral Child DevelopmEnt Studies (INCLUDE) that focus on different aspects of the child’s development in context of social, migration and trauma background in refugee and non-refugee clinical populations by not specifically drawing a borderline between refugee and non-refugee children, but rather taking advantage of multiple parameters exercised in the project.

### Study population

The study population consists of 70 children with refugee experience (RG) and 111 children born and raised in Germany as comparison group (CG), all aged between 3 and 6 years. The data of the refugee children were collected between June 2021 and August 2022 in two Munich refugee camps in Fürstenfeldbruck and Am Moosfeld. Parents were contacted in our interdisciplinary consultations and almost all of the families joined to participate in the study. The families in the RG mostly arrived from Afghanistan (*n* = 59, 84%), three children from Congo and Venezuela (4% each), one child each from Bolivia, Yemen, Palestine, Sierra Leone, and Uganda (1% each). Informed consent was obtained from all parents and children. The study protocol was approved by the ethics committee of the Medical Faculty of the Technical University of Munich. The data of the CG were collected between January and April 2022 in the Social Pediatric Center (SPZ – Sozialpädiatrisches Zentrum) of the kbo-Kinderzentrum in Munich, Germany, where the children were referred due to developmental, language and/or social-emotional difficulties [33]. In the CG, 86 of the children (78%) had at least one parent who was not born in Germany. After clinical examination, 34 children (31%) were diagnosed with a combined developmental disorder (F83) and 39 (36%) with a speech or motor developmental disorder (F80, F82), and one child received a high-functioning Asperger diagnosis. A psychiatric diagnosis (F43, F51, F90-F98) was given to 50 children (44%). Children with an expert-diagnosed autism spectrum disorder, intellectual impairment, specific syndromes or other serious neurological or psychiatric disorders have been excluded from the study.

For a more detailed description of the sample see Bernhardt et al. 2023 [33].

### Measures

We assessed parental education by the total years of education of both parents (if available) divided by two. Parental distress has been operationalized by the “Refugee Health Screener” (RHS) [34] which comprises various psycho-somatic issues of the parents, like anxiety, pain, somatic, posttraumatic and depressive symptoms, and additionally takes into account the parental level of stress, visually represented on a 10-point scale. We calculated a sum score capturing both issues [21].

In order to objectify the child’s distress, the level of cortisol was measured in ng/ml in the saliva, referring to protocols suggested by Adam & Kumari [24]. After detailed instructions by the staff, the collection was done by the parents three times a day (morning directly after wake-up, afternoon, evening right before bedtime) on a normal weekday in the home environment. Focusing on the decline of cortisol towards the evening and mitigating the effect of high intraindividual cortisol variations during the cortisol awakening response, we included a sum score of afternoon and evening cortisol level in our analyses to investigate if the amount of screen exposure correlates with a failure of physiological circadian cortisol decline towards the second half of the day [21].

The children’s cognitive, learning and language performance have been operationalized by the non-verbal IQ (Scale of Intellectual Functioning, SFI) and the subtests Atlantis and Vocabulary (only in CG) of the Kaufman Assessment Battery for Children (KABC-II), a neuropsychologically developed instrument for assessing the processing and problem-solving abilities of children and adolescents between 3 and 18 years of age in a culture-fair manner [35]. The test is reported to give special attention to use with handicapped groups and children with learning disabilities, as well as being appropriate for cultural and linguistic minorities. The memory-task (subtest Atlantis) to assess the children’s short-term learning performance by memorizing nonsense names for pictures of fishes, plants and shells (the child is requested to point to the correct picture when the instructor reads out the names) shows low intercorrelations with the general cognitive scale (0.28) and a high reliability (0.97), independent of the parents’ migration status [35]. With low correlations with tests for short-term selective attention ( −  0.06 SIF,  – 0.02 Atlantis) both tasks show good discriminant validity [35]. In the subtest Vocabulary children are asked to name pictures of objects [35].

Educators in kindergarten or school were asked to rate the child’s German language skills on a self-constructed 5-point scale with 0 meaning the child doesn’t understand or speak any German at all and 5 meaning the child understands and speaks German age-adequately and like a native speaker.

The amount of screen exposure has been assessed in parent rating by asking parents about their child’s total screen exposure per day with open questions. Answers were categorized in three groups (ordinally scaled):

$${\text{1}} = \, < \,1~hours/{\text{ }}day;{\text{ }}2\, = \,1{-}2~hours/{\text{ }}day;{\text{ }}3\, = \,\, > \,2~hours/{\text{ }}day$$

With setting the lower limit at one hour and the upper limit at two hours per day, the classification system represents the divisions of groups according to expert recommendations (less than one hour/day) on the one hand [6, 7, 36] and the current average of media use in pre-school children (more than two hours/day) on the other hand [1].

### Statistical procedure

In advance to further analyses we calculated independent t-tests to compare the CG and FG in the assessed demographic and test variables (age, parental education and distress, learning, non-verbal IQ, salivary cortisol). For the analyses concerning our research question we used nonparametric Spearman correlations to quantify relations of screen exposure with socio-demographic variables and estimates for language development. Concerning salivary cortisol levels we used the non-parametric Mann–Whitney-U- and Kruskal–Wallis-Tests for independent samples to assess effects of screen exposure separately for the refugee and the comparison group. To compare the groups concerning learning and cognitive performance with regard to their amount of screen exposure we performed a multifactorial analysis of covariance (MANCOVA) by adding the parental education as a covariate. Post-hoc analyses for subgroup comparisons were Bonferroni-corrected.

## Results

We first describe demographic and contextual variables and correlations to the amount of screen exposure (Sects. Demographics, Screen time and Social context factors of the results section), respectively, and then relate it to biological stress (Sect. Biological stress level) and cognition, learning and language assessment as markers of development (Sect. Impact on development).

### Demographics

For a description of the study population see Table 1.

**Table 1:** Characteristics of the sample

		Total	CG	RG
Male	N (%)	115 (64)	79 (71)*	36 (51)*
Age	N	181	111	70
	Mean (SD)	5.10 (1.11)	5.20 (1.08)	4.94 (1.16)
	Range	3.00 – 6.97	3.01–6.97	3.00–6.93
Parental education (years)	N	174	107	67
	Mean (SD)	9.40 (5.23)	11.55** (3.36)	5.97** (5.83)
	Range	0–20	1–20	0–19.5
Parental distress (sum score)	N	171	106	65
	Mean (SD)	120.03 (156.58)	43.27** (76.47)	245.20** (172.79)
	Range	0–583	0–462	4–583

*P < 0.05; **P < 0.001

The groups did not differ in age (t(179) = 1,526, *p* = 0.129; 95%-CI [ – 0.08, 0.59]), while the CG had a significantly higher percentage of boys (71,2%) than the RG (*X*^*2*^(1) = 7.22, *p* = 0.007). There were significant differences between the groups in years of parental education (t(93,909) = 7,140, *p* < 0.001; 95%-CI [4.03, 7.14]) and parental distress (t(79,599) =  – 8,903, *p* < 0.001; 95%-CI [ – 247.07,  – 156.79]) with the RG-parents having less formal education and reporting more distress than the CG.

As depicted in Table 2, the groups did not differ in cumulated afternoon and evening cortisol (t(71) =  – 0.974, *p* = *0.333;* 95%-CI [ – 4.51, 1.55]), but in learning performance (t(166) = 5,494, *p* < 0.001; 95%-CI [1.64, 3.48]) and IQ-test-scores (t(160) = 3,258, *p* = 0.001; 95%-CI [3.52, 14.35]) with the RG scoring significantly lower than the CG on both measures. For IQ-test-scores, group differences diminished when the amount of parental education was added as a covariate (see Sect. Learning and cognitive development).

**Table 2:** Test results in CG and RG

		Total	CG	RG
Salivary cortisol (cumulated afternoon & evening)		73	39	34
		6.45 (6.48)	5.76 (5.58)	7.24 (7.38)
		0.99–36.50	1.67–29.60	0.99–36.50
Learning (subtest Atlantis)	N	168	102	66
	Mean (SD)	7.98 (3.19)	8.98** (3.13)	6.42** (2.63)
	Range	2–17	2–17	2–12
Non-verbal IQ^1^ (KABC-II, SFI)		162	101	61
		86.67 (17.4)	90.03** (17.35)	81.10** (16.15)
		46–126	46–125	55–126
Language (subtest Vocabulary)		NA	100	NA
			6.18 (3.29)	
			0–14	

*P < 0.05; **P < 0.001

^1^The IQ of the comparison group was assessed using either the KABC-II (N = 61) or a comparable standardized nonverbal IQ test such as Wechsler Preschool and Primary Scale of Intelligence – Fourth Edition (WPPSI-IV; N = 38), Wechsler Nonverbal Scale of Ability (WNV; N = 1), or Snijders-Oomen Nonverbal Intelligence Test (SON-R 2–8; N = 1)

### Screen time

**Fig. 1:**
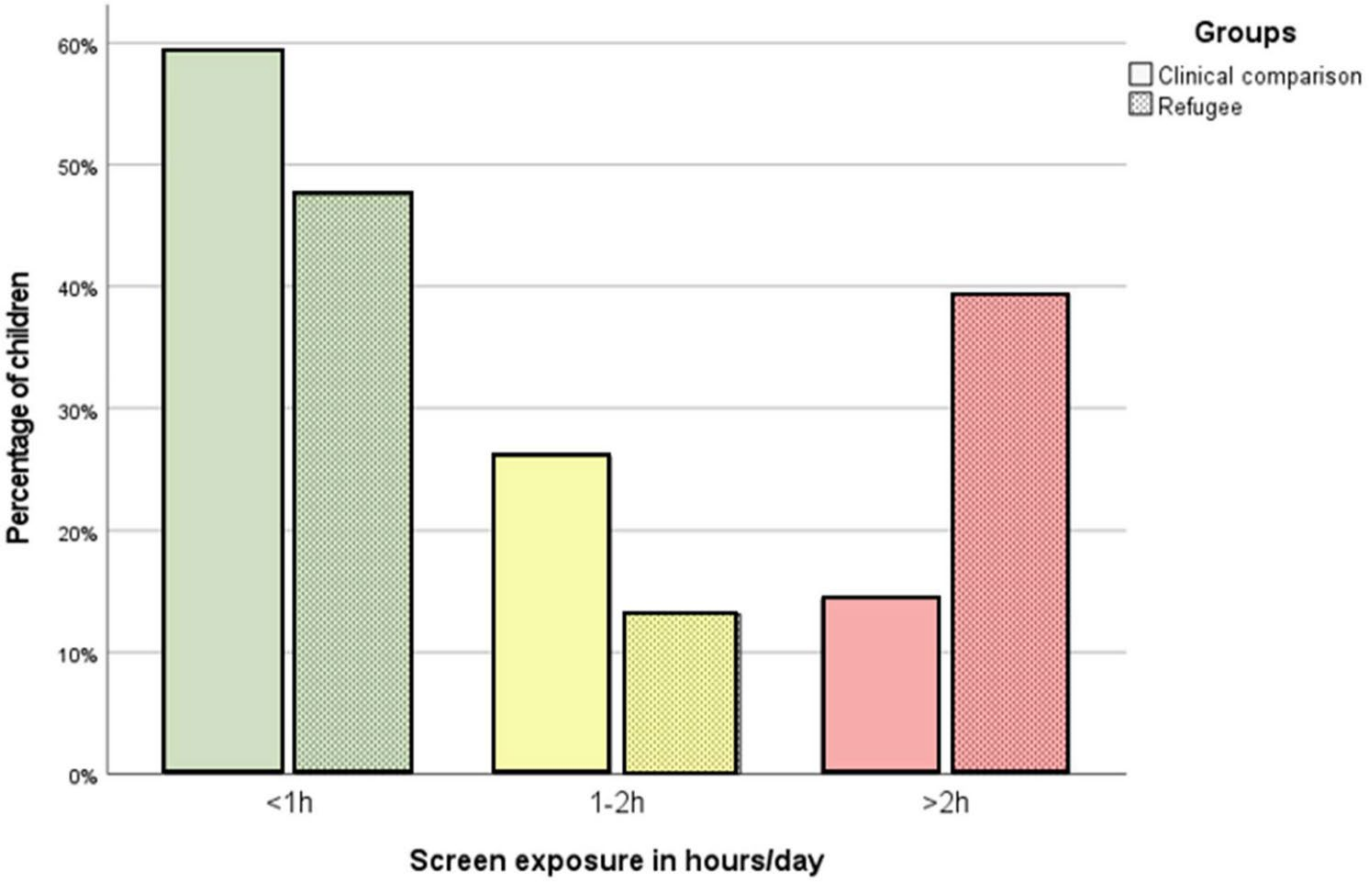
Screen exposure in hours per day in CG and RG

In the overall study population, 95 children (55%) are reported to be exposed to electronic screen media for less than one hour per day and 40 (23%) for more than two hours per day. As depicted in Fig. 1, we notice significantly differential distributions in the two subgroups (*U*_*CG-RG*_ = 2698, Z =  – 2.446, *p* =  < 0.05, n = 172): While among the children in the clinical comparison group it’s 14% (16 children) referring more than two hours screen exposure per day, the corresponding rate in the refugee group is 39% (24 children). In the CG, the distribution of diagnoses for developmental delays or psychiatric disorders (ICD10-codes) did not differ with respect to the amount of reported screen exposure (*p* = 0.31 for developmental delay*, p* = 0.71 for psychiatric disorders).

### Social context factors

The data reveal a significantly negative correlation in the whole sample between the parents’ education and the child’s screen exposure (r_edu_ =  – 0.194; *p* = 0.012; n = 166). The parents’ distress level is significantly positive correlated to the child’s screen exposure (r_rhs_ = 0.238; *p* = 0.002; n = 165).

### Biological stress level

**Fig. 2:**
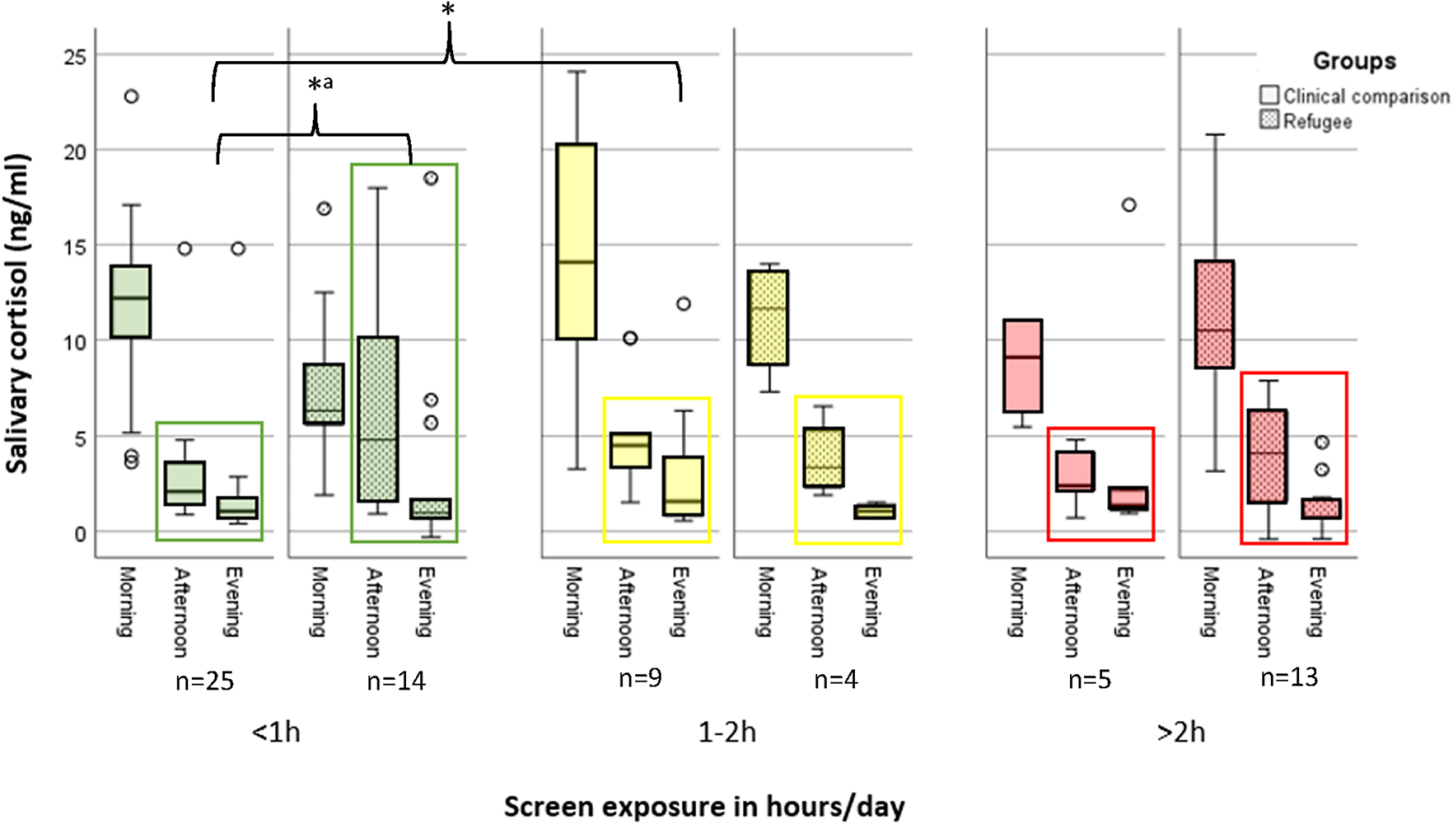
Salivary cortisol during day-time in different categories of screen exposure in CG and RG

When comparing rank-scaled cumulated afternoon-evening-levels of salivary cortisol for the different amounts of screen exposure within the two groups, we find a higher amount of screen exposure to be associated with significantly higher values of salivary cortisol in the CG (H_CG_ (2 df) = 8.841, *p* = 0.012), whereas we cannot detect any corresponding significant effect in the RG (H_RG_ (2 df) = 1.433, *p* = 0.49), see Fig. 2.

Correspondingly, only in the CG we find a significantly positive correlation between the amount of screen exposure and the cumulated cortisol levels of the afternoon and evening (r_Cortisol-CG_ = 0.405, *p* = 0.01, n = 39). Comparing cumulated afternoon and evening cortisol between RG and CG in different categories of screen exposure yielded a statistically significant group difference only within the condition of screen time less than one hour/day (*U*_*2G*_ = 110, *Z* =  – 1.903, *p* = 0.03, one-tailed).

### Impact on development

#### Learning and cognitive development

**Fig. 3:**
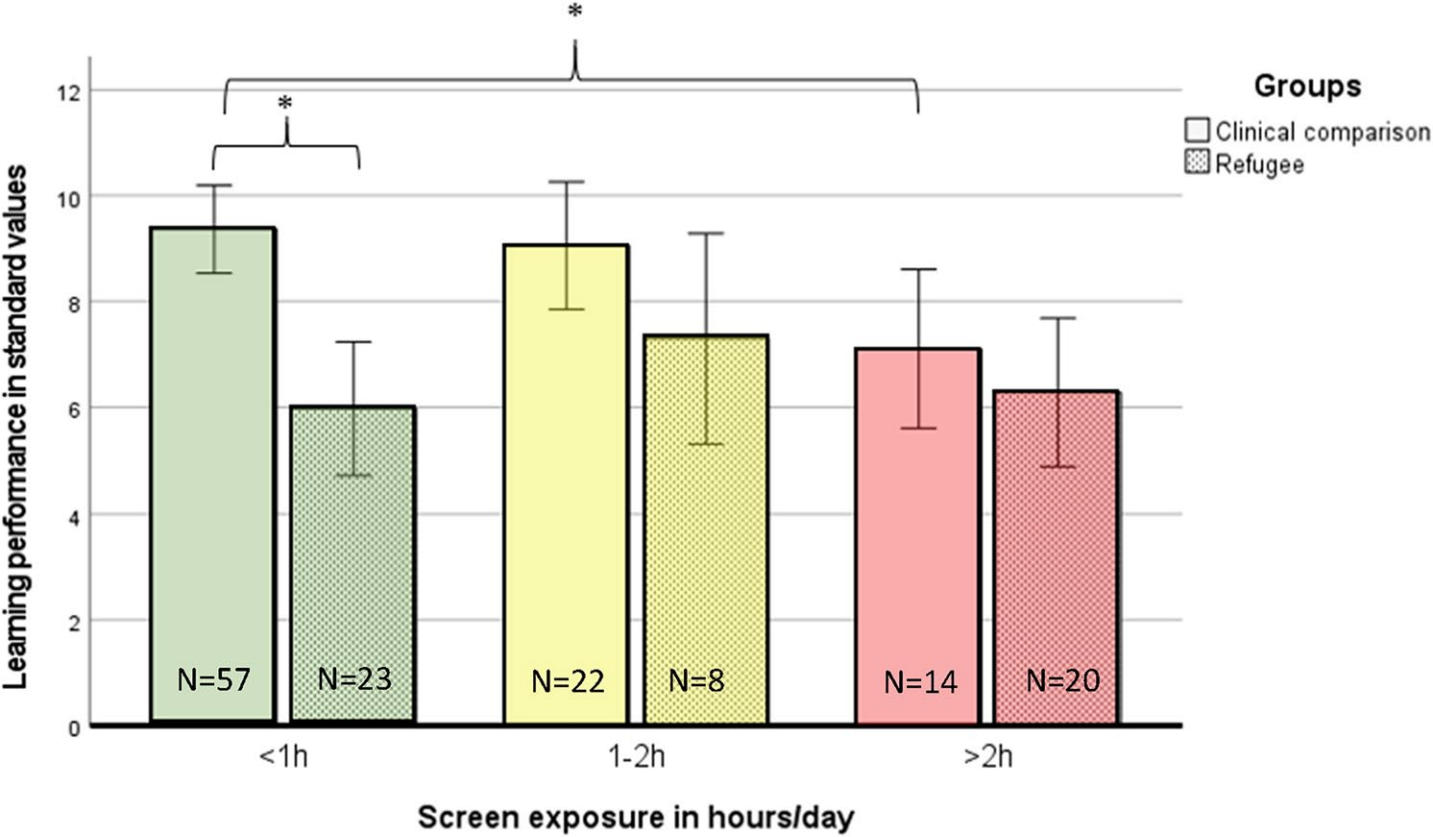
Learning performance in different categories of screen exposure in CG and RG

When performing a MANCOVA with learning performance and IQ-test-scores as dependent variables, clinical comparison and refugee group as group variables and the parents’ education as a covariate, we detect a significant group main effect for learning performance with the refugee group showing a lower learning performance than the clinical comparison group (F_Atlantis-Group_ = 8.373 (1, 137), *p* = 0.004) and a significant effect of screen exposure on learning performance within the clinical comparison group (F_Atlantis-CG_ = 3.407 (2, 137), *p* = 0.036) with pairwise post-hoc-tests revealing significant differences for learning performance between amounts of screen exposure less than one and more than two hours per day (*p*_<1 h/d__vs_._>2 h/d_ = 0.031). Further, as depicted in Fig. 3, the CG and the RG differ in learning performance only when the children’s screen exposure is less than one hour per day (F_<1 h/d_ = 16.447 (1, 137), *p* < 0.001).

For higher amounts of screen exposure, there are no group differences, as the learning performance of the comparison group equals the lower sub-standard level of the refugee group (F_1-2 h/d_ = 2.098 (1, 137), *p* = 0.150; F_>2 h/d_ = 0.346 (1, 137), *p* = 0.557). No corresponding significant results could be detected for the IQ-test scores.

#### Vocabulary and language acquisition

The CG shows sub-standard performance on the subtest Vocabulary of the KABC-II (see Table 2), which is not correlated to the amount of screen exposure (r_V-SE-CG_ = -0.195, *p* = 0.05; n = 100). For educator rating of the child’s German language skills within this group we find a significantly positive correlation with the standardized subtest Vocabulary (r_German-V-CG_ = 0.615, *p* < 0.001; *n* = 49) as well as a significantly negative correlation with the amount of screen exposure (r_German-SE-CG_ =  – 0.270, *p* < 0.05; *n* = 54). For our whole study population, the educator rating of the child’s German language skills demonstrates a significantly negative correlation with the amount of screen exposure (r_German-SE-total_ =  – 0.251; *p* = 0.04; *n* = 67).

To summarize the results in total, we present the correlation matrix for the relevant variables in Table 3.

**Table 3:** Correlation matrix of the sample

	Parental education	Parental distress	Salivary cortisol	Learning	Non-verbal IQ	Vocabulary	German language
(a) *Correlation matrix for the total study population*							
Screen time	– 0.194*	0.238*	0.124	– 0.176*	– 0.015	– 0.186	– 0.251*
Parental education	1	– 0.316**	– 0.079	0.259**	0.204*	0.354**	0.367*
Parental distress		1	0.122	– 0.187*	– 0.118	– 0.047	– 0.218
Salivary cortisol			1	0.006	– 0.021	– 0.107	0.042
Learning				1	0.566**	0.486**	0.472**
Non-verbal IQ					1	0.447**	0.415**
Vocabulary						1	0.615**
(b) *Correlation matrix for CG*							
Screen time	– 0.204*	0.070	0.405*	– 0.190	– 0.022	– 0.195	– 0.270*
Parental education	1	0.183	– 0.321	0.141	0.282*	0.350**	0.269
Parental distress		1	– 0.073	0.092	0.217*	– 0.023	0.169
Salivary cortisol			1	0.045	0.032	– 0.107	– 0.056
Learning				1	0.490**	0.487**	0.201
Non-verbal IQ					1	0.445**	0.277*
Vocabulary						1	0.615**
(c) *Correlation matrix for FG*							
Screen time	0.019	0.214	– 0.198	0.043	0.160	–	– 0.608*
Parental education	1	– 0.012	0.117	0.034	– 0.071	–	0.275
Parental distress		1	0.118	0.097	– 0.096	–	– 0.465
Salivary cortisol			1	0.170	0.037	–	0.507
Learning				1	0.590**	–	– 0.461
Non-verbal IQ					1	–	– 0.304

* *p* < 0.05, ** *p* < 0.001, Pearson-Correlation for parental education, parental distress, salivary cortisol, vocabulary; Spearman-Correlation for screen time, German language in educator rating

## Discussion

This study uses an experimental approach by examining children with and without refugee experience (and not solely relying on parental rating) to relate the amount of screen exposure in the home environment to biological stress and highly relevant developmental aspects in vulnerable populations. In our sample with combined risk factors, a group which is underrepresented in research on effects of screen exposure so far, the children’s amount exceeds expert recommendations and is significantly related to parents’ level of education and distress. Only in the condition of less than one hour of screen time per day, the CG shows age-appropriate biological stress regulation and learning performance that differs significantly from the RG’s scores. In the condition of more than one hour of screen time per day, both groups show equally weak cortisol down-regulation towards the evening and in the condition of more than two hours of screen time per day both groups show low learning performance. Correspondingly, we found a meaningful and significantly negative correlation between language performance and the amount of screen exposure in our sample, whereas there is no significant relation to non-verbal IQ-test performance.

With these results we replicate earlier findings that children in high-stress-contexts show elevated cortisol levels and lower learning performance [21].

One option to explain these findings could be the children’s reduced attention span due to limited opportunities for social interaction and less practice in tasks that demand a joint attention focus in the testing situation [18].

As in this study the IQ-test-performance is not correlated to screen exposure and the refugee children show equally weak cortisol down-regulation towards the evening and low learning performance in all conditions of screen exposure, we consider the cumulative stress as the most plausible explanation: Whereas the refugee children suffer from high stress in all categories of screen exposure, the elevated screen exposure might be the critical element for the children in the clinical comparison group to increase their stress hormones to a level where cortisol impairs learning [21, 25, 37–40].

The positive correlation between parental distress and the infant’s amount of screen exposure is in line with previous findings that parents who feel more burdened show less sensitivity in their interactions with the child [41, 42] and thus might be more susceptible to calm or distract their child with electronical devices. As a consequence, less time spent in interactions and communication further limits the opportunity for reciprocal imitation, which is one of the most important prerequisites of language acquisition [43, 44].

### Strengths

One of the strengths of the study is the sample with combined risk factors, reflecting a population that is often neglected in the general discourse and less visible in research projects. With our group of refugee and help-seeking families we are not subjected to ceiling effects for parental education like several other studies investigating children’s development and corresponding environmental conditions, as studies that rely on voluntary participation are more likely to involve only higher educated subgroups of the population [10, 45, 46]. As in our clinical CG 78% of the parents had a migration background, families in both groups share characteristics like multilingual environments and relocation experiences.

With our multi-rater and multi-method-approach we do not rely solely on parent rating (that might be skewed by parents’ expectations and social desirability), but also on child examinations with biologically (salivary cortisol) and psychologically (learning and language performance, non-verbal IQ) objectively measured outcomes as well as educators’ expert ratings (language performance).

### Limitations

Although our results show that social context factors like parental education and distress are related to children’s media consumption habits, our cross-sectional design does not allow to distinct determinants that cause high screen exposure from those which are negatively influenced by it. But we have to state that we do not find any hints in our data that children that are less intelligent, more delayed in their development or more symptomatic tend to be exposed to screen media more often.

Further, the RHS as a questionnaire has been developed with and for people with flight and refugee experience and might not be appropriate to measure stress in a non-refugee-population. But nevertheless, this instrument offers a feasible and low-threshold way to assess parental distress in diverse populations that might have experienced potentially traumatic events. As the content of the questions is conveyed in easy language and answers are depicted with symbols, we see this questionnaire as an appropriate assessment in our sample, where not only the RG but also the majority of families in the CG group had a migration background and multilingual environment.

Along with the majority of research studies on young children’s screen time in everyday life that rely on parent report [47], the amount of screen exposure in this study has been reported by parents and not by objective means, capturing duration only and not assessing information on device, type, content and context of media use (as recommended by Byrne et al. [47]). Referring to social desirability and considering that the data collection took place during the covid pandemic where most group activities were restricted, we might rather have underestimated the real exposure time, which further highlights the relevance of our results. The covid pandemic within the last years has not only limited access to group and educational facilities that offer distraction and ideas for non-screen occupation for children and caregivers, but also augmented parental stress symptoms [45, 46].

As a suggestion for further research we recommend to not solely rely on parent ratings concerning screen time, but also consider the context of screen use and supplemental information from the child’s assessment that point to excessive use (e.g. amount of time spent in day-care, apps on children’s devices documenting screen-time etc.) and to consider parents’ screen times and longitudinal data to disentangle cause and effect. As algorithms designed to extend the duration and addictiveness to digital screen contents are constantly developing, prompt assessments to capture the complexity of children’s real-time screen exposure are necessary and need to be developed [47].

## Conclusion

The combination of adverse psycho-social aspects increasing parental burden and low parental education are associated with higher amounts of screen exposure in children, which are in turn correlated to elevated biological stress and lower learning and language performance in a clinical comparison group from a help-seeking population. As especially early learning and language acquisition problems are likely to cause difficulties later in life, it must be suspected that the full degree of the developmental disorders is yet to come. We call for strict implementation of expert recommendations concerning screen exposure in young children in clinical routines and thorough education for parents.

## Data Availability

Data is made available on reasonable request.
