# Correction: Correlation of screen exposure to stress, learning, cognitive and language performance in children

**DOI:** 10.1007/s00787-024-02625-1

**Published:** 2024-12-09

**Authors:** Andrea Hahnefeld, Monika Fink, Saskia Le Beherec, Marie Anna Baur, Katharina Bernhardt, Volker Mall

**Affiliations:** 1 Chair of Social Pediatrics, TUM School of Medicine, Technical University of Munich, Munich, Germany; 2 Kbo Kinderzentrum, Heiglhofstrasse 65, 81377 Munich, Germany; 3 German Center for Child and Adolescent Health (DZKJ), Partner Site, Munich, Germany

**Correction: European Child & Adolescent Psychiatry** 10.1007/s00787-024-02593-6

In the original version of this article, Andrea Hahnefeld and Monika Fink were denoted as joint first authors only in section Author contributions.

The original article has been corrected: Joined authorship additionally mentioned on first page.

